# Study on the Fabrication Process of X-ray Focusing Mirrors

**DOI:** 10.3390/mi14091666

**Published:** 2023-08-26

**Authors:** Qiuyan Liao, Fei Ding, Zhigao Chen, Duo Li, Bo Wang

**Affiliations:** College of Mechanical Engineering, Harbin Institute of Technology, Harbin 150001, China; liaoqiuyanln@163.com (Q.L.); liduo@hit.edu.cn (D.L.)

**Keywords:** fabrication process, diamond-like carbon (DLC), release layer, X-ray focusing mirrors, demolding device

## Abstract

The eXTP (enhanced X-ray Timing and Polarization) satellite is a prominent X-ray astronomy satellite designed primarily for conducting deep space X-ray astronomical observations. The satellite’s scientific payload consists of X-ray focusing mirrors. In order to fulfill the requirements of weight reduction and enhanced effective area, the thickness of mirrors is reduced to the sub-millimeter range and a multi-layer nested structure is employed. Manufacturing mirrors poses a significant challenge to both their quality and efficiency. The present research investigates the optimal replication process for mandrel ultraprecision machining, polishing, coating, electroforming nickel, and demolding. It analyzes the factors contributing to the challenging separation and the inability to release the mirror shells. Additionally, an automatic demolding device is developed, and the X-ray performance of the replication mirrors is verified. The fabrication process flow of the mirrors was initially introduced. To ensure the easy release of the mirror shells from the mandrels, a layer of diamond-like carbon (DLC) was applied as a release layer between the Au and NiP alloy. The adhesion strength of Au-C was found to be significantly lower than that of Au-NiP, as demonstrated by both molecular dynamic simulation and tensile testing. The development of an automatic demolding device with force feedback has been successfully completed. The reduction in the half-power diameter (HPD) of the mirror from 48 inches to 25 inches is an improvement that surpasses the production target.

## 1. Introduction

The production of X-ray focusing mirrors falls under the category of extreme manufacturing. This process typically involves achieving accuracy within the nanometer range on a scale of meters and achieving surface roughness below one nanometer. X-ray optics play a crucial role in the domain of space-based high-energy ray detection, catering to a wide range of extensive and time-sensitive application requirements. The eXTP satellite—its full name is enhanced X-ray Timing and Polarimetry Mission space observatory [1,2]—for example, that will be launched in 2027 is the largest international cooperative space science project initiated and led by Chinese scientists, and also the next-generation flagship X-ray astronomical satellite. The development stage of the eXTP satellite has encountered a significant challenge in fully breaking through the manufacturing technology of the X-ray focusing mirror. The platform incorporates a total of 13 sets of X-ray focusing telescopes. Each set comprises 50 mirrors, with a maximum aperture of approximately φ500 mm and a length of 600 mm. The focusing mirror exhibits a surface roughness of less than 0.5 nm RMS, while the overall surface shape accuracy surpasses 0.1 μm PV. The current optical manufacturing technology is faced with a significant challenge due to this situation.

**Figure 1:**
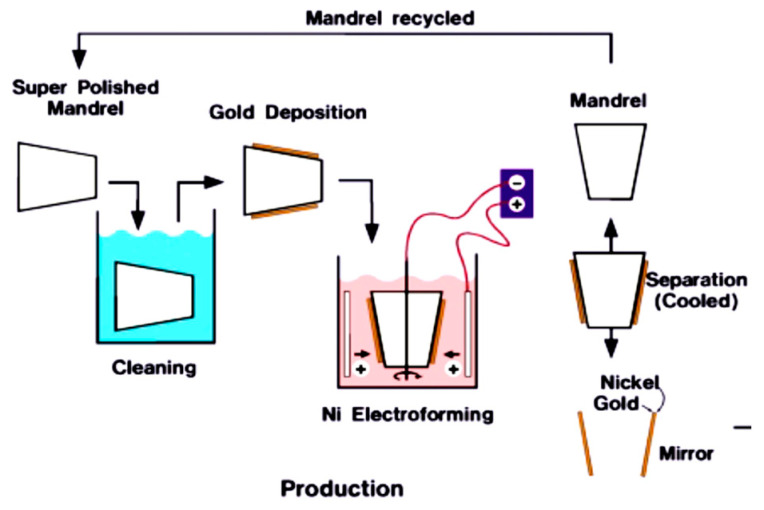
Steps of the replication process of the electroforming replication method.

Since the 1970s, numerous research institutions worldwide have conducted extensive research on the manufacturing technology of the nesting Wolter-I-type grazing incident X-ray focusing mirror. The primary manufacturing processes that have been developed include direct polishing [3], wafer splicing [4,5], glass sheet thermal collapse [6], and electroforming replication [7,8,9,10,11,12]. Currently, the prevailing manufacturing process for international X-ray optical systems relies on the utilization of ultraprecision mandrel electroforming nickel reproduction process technology. The concept of this technology was initially introduced in the early 1990s and later implemented on the XMM-Newton satellite, which was successfully launched in December 1999 [13]. Since then, more than 10 related satellites launched or planned to be launched by ESA and NASA [14,15,16,17,18,19] have adopted such technology. The electroforming copying method has emerged as the most technologically mature manufacturing process for X-ray focusing mirrors when considering factors such as manufacturing efficiency and accuracy. Figure 1 depicts the flowchart illustrating the electroforming replication technique employed for the production of Wolter-I focusing mirrors [20].

China’s involvement in the manufacturing of X-ray optical systems began relatively late, resulting in a significant lag in the development of high-resolution and large effective area technologies. As a consequence, the production of these optical systems is currently reliant on foreign procurement. This dependence on external sources has implications for the X-ray optical system of China’s planned eXTP satellite, as it is subject to the involvement of European and American entities. In addition, in the fields of pulsar navigation, divine light engineering, space-based early warning, and advanced light sources, they are also facing extreme manufacturing technology bottlenecks in X-ray optical systems. To be able to mitigate the foreign technology monopoly and minimize potential schedule risks, it is imperative for China to promptly undertake independent research and development of X-ray optical system manufacturing technology. This will enable China to establish autonomous and controllable core technology. To gain a comprehensive understanding of the autonomous and controllable process of nickel electroforming replication, it is imperative that we undertake an exploration of this procedure independently. Ultimately, the space X-ray detection and pulsar navigation research area will benefit from the X-ray mirrors.

The electroforming nickel copying process is commonly employed due to its numerous benefits, including high precision, low cost, and a stable process. However, the bond between the X-ray focusing mirror and the electroforming nickel die is robust, making demolding challenging. Consequently, a certain level of technical expertise and experience is necessary to overcome this issue [21]. In order to solve this problem, NASA uses passivation [22] on the surface of the mandrel, and other institutions use the method of adding TiN [23,24] isolation film to reduce the binding force between the mandrel and the shell, but the specific details have not been reported. Due to its excellent adhesion resistance and mechanical properties, DLC film becomes one of the choices of isolation film; finally, it is verified in this paper.

This paper presents the following contributions. The autonomous and controllable process of nickel electroforming replication was developed to produce X-ray focusing mirrors efficiently and reliably. The investigation of the correlation between interfacial energy and binding force was conducted through the utilization of molecular dynamics calculation and tensile testing. Then, in order to release the mirror shells easily from the mandrels, we added the DLC as a release layer between Au and NiP alloy. A force-feedback automatic demolding device has been developed to ensure the replication principle of first separation and then lifting. This device aims to achieve high-quality production of X-ray focusing mirrors.

## 2. Efficient Replication Process Route

### 2.1. Chemical Plating with Nickel–Phosphorus Alloy

The electroless nickel–phosphorus alloy layer exhibits a phosphorus content of approximately 10% in an amorphous structure. This composition demonstrates excellent performance in ultraprecision machining, allowing for the achievement of surface roughness levels below 1 nm. Before electroless nickel plating for the aluminum alloy mandrel of the X-ray focusing mirror, the surface roughness should be roughed first, and then electroless nickel plating conducted. After electroless nickel plating, the Ni-P alloy layer should be ultraprecision machined to achieve the surface profile error <1 μm and surface roughness RMS <1 nm. Electroless nickel plating necessitates meeting several stringent requirements. Firstly, it is imperative that the coating exhibits a strong adhesion to the aluminum alloy matrix. Secondly, the coating must possess a phosphorus (P) content exceeding 10% to guarantee an amorphous structure and uniform composition. Additionally, the coating should be free from any defects. Lastly, the desired coating thickness should measure 150 μm.

### 2.2. Ultraprecision Machining of the Mandrel

**Figure 2:**
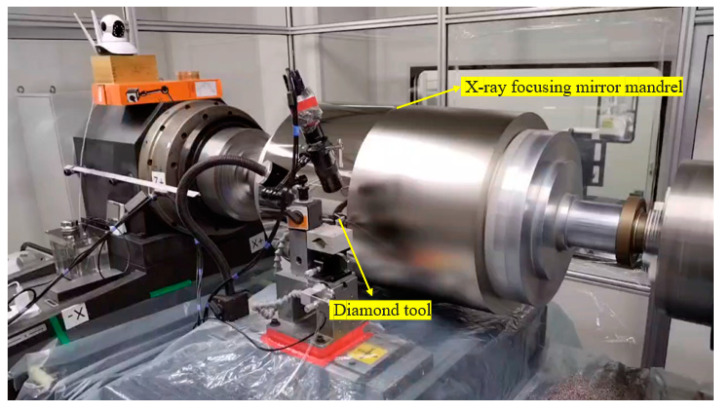
X-ray focusing mirror mandrel ultraprecision machining process.

The precision of an ultraprecision machine tool, which serves as the primary equipment for processing focusing mirror mandrels, has a direct impact on the shape accuracy and surface roughness of the resulting focusing mirror mandrels. The processing range and accuracy of the existing commercial ultraprecision turning equipment cannot meet the processing requirements of the focusing mirror mandrel. Hence, we have developed an autonomous design for a high-capacity horizontal mandrel ultraprecision machining machine. This machine incorporates a three-axis linkage layout scheme, specifically, X-Z-C, to ensure optimal performance. Among them, the X and Z axes of the machine tool are linear motion axes, which are supported by hydrostatic pressure and driven by linear motor. The Z-axis grating resolution is 1 nm, and the X-axis grating resolution is 5 nm. The C-axis refers to a spindle used for holding workpieces. It offers accurate control over angular movement and is supported by aerostatic pressure. The spindle is driven by a frameless torque motor and has a circular grating resolution of 0.36 inches. The tailstock spindle seat has the capability to move in the Z-axis direction, allowing it to accommodate the processing needs of varying lengths of focusing mirror mandrels. The maximum length of the mandrel that can be processed is 2200 mm, and the maximum straight is 600 mm. The four-jaw compound chuck is installed at the main shaft of the head frame and the main shaft of the tail frame. The chuck has the function of self-centering and can also adjust each jaw separately to realize the precise in-place adjustment of the roll die. The efficient fabrication of high-precision mandrels relies on the utilization of ultraprecision turning techniques. This involves employing natural single-crystal diamond tools and taking advantage of the inherent precision of ultraprecision machine tools. By doing so, it becomes possible to effectively process the mandrel with nano-level surface roughness and submicron-level surface shape accuracy. This capability is crucial for achieving efficient and accurate fabrication of high-precision mandrels. The primary shaft operates at a rotational speed of 360 revolutions per minute (rpm), while the feed speed is 2 μm per revolution (μm/r). Additionally, the cutting depth is set at 2 μm (μm). Figure 2 illustrates the ultraprecision machining process.

### 2.3. Ultra-Smooth Polishing

**Figure 3:**
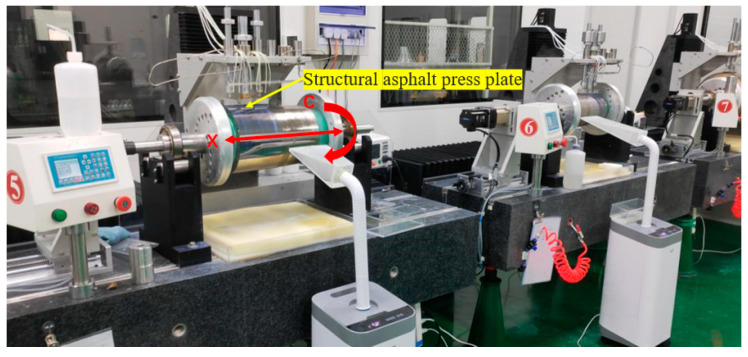
Ultra-smooth polishing process.

Despite its ability to achieve nanoscale surface roughness, ultraprecision turning falls short in meeting the reflection requirements of X-ray wavelengths. Additionally, the resulting turned surface exhibits uniform thread-like tool marks, leading to significant scattering of incident light. Therefore, after the ultraprecision turning process, the ultra-smooth polishing process must be used to remove the cutting lines on the turning surface and further improve the surface quality. The development of a full-size ultra-smooth polishing device was achieved independently. This device was utilized in conjunction with a validated ultra-smooth polishing process. A self-manufactured structural press plate served as the polishing tool, while asphalt and a damping polishing cloth were employed as the polishing materials. The polishing liquid consisted of 20 nm silica sol. The polishing process is depicted in Figure 3.

### 2.4. Mandrel Coating and Nickel Electroplating

**Figure 4:**
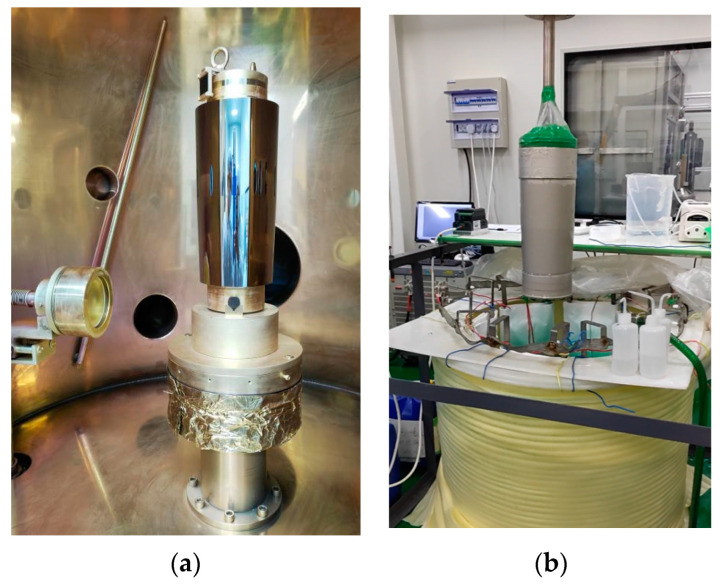
Coating (**a**) and electroforming (**b**).

Following the cleaning process, the mandrel undergoes vacuum plating with an Au film. A layer of gold, approximately 100 nm in thickness, is sputtered onto the polished surface of the mandrel. This sputtering process is carried out using RF magnetron sputtering equipment, as depicted in Figure 4a. The electroforming process involves the formation of a hundred-micron nickel layer on the mandrel’s surface after coating. This results in the creation of an independent shell structure. Electroforming is made use of for cathode deposition in the processing of electrochemical electrodes. The procedure involves utilizing the mandrel as the cathode; the electroforming material as the anode; and the electroforming solution, which comprises the metal salt solution containing the electroforming material. When subjected to direct current (DC) voltage or pulse current, the metal ions present in the electroforming solution undergo reduction at the cathode, resulting in the formation of metal atoms. These metal atoms are subsequently deposited onto the surface of the mandrel. Simultaneously, the metal at the anode undergoes continuous transformation into ions and dissolves into the electroforming solution. This process serves to replenish the metal ion concentration in the solution, thereby maintaining its overall stability. In the electroforming process, it is necessary to automatically control the temperature, monitor the pH value of the bath, and adjust it in time. At the same time, the stress of the coating is measured by the test plate, and the current density is adjusted in a timely manner. The control of the electroforming nickel shell’s thickness is based on the level of electricity supplied by the DC power source. The electroforming process is halted once the predetermined electricity level, as depicted in Figure 4b, is attained.

### 2.5. Demolding

**Figure 5:**
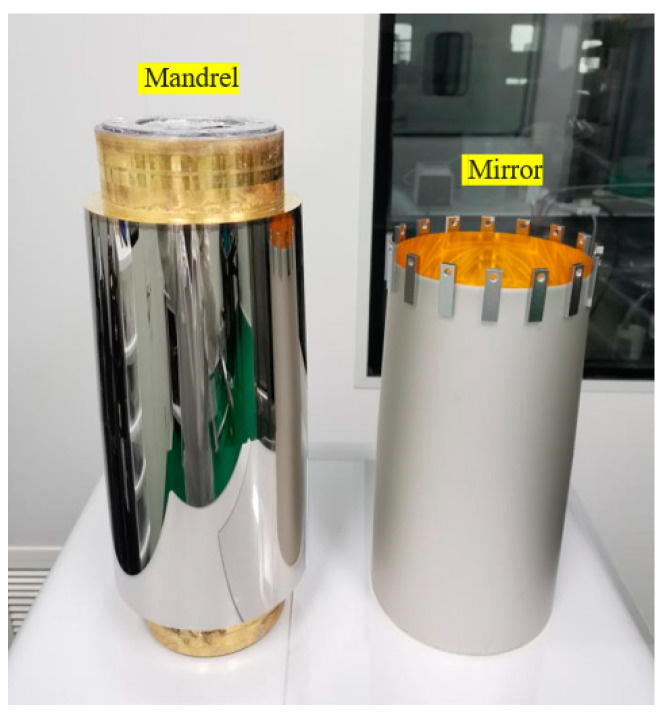
Mandrel and mirror after demolding.

The demolding process represents the final stage in the X-ray fabrication process chain and is considered the utmost crucial step in ensuring quality assurance. The mandrel is detached from the gold film nickel matrix mirrors, and the inner surface of the gold film reflector is used as the X-ray reflector. After demolding, the high-precision focusing mirror mandrel can be recycled, which improves manufacturing efficiency and reduces production cost. The fundamental concept behind mandrel release involves the introduction of liquid nitrogen into the mandrel’s hole. This process effectively cools down the mandrel and nickel shell, causing them to undergo elastic contraction due to the low-temperature conditions. The thermal expansion coefficient disparity between the nickel shell and the aluminum mandrel results in a greater contraction degree of the mandrel matrix compared to the outer nickel shell. This discrepancy in contraction stress on both sides generates a higher contraction stress, effectively nullifying the interface radial binding force between the mandrel and the nickel shell. Consequently, this allows for the radial separation of the mandrel and the mirrors. Upon completion of the demolding process, the mandrel and mirror exhibit the configuration depicted in Figure 5.

## 3. Molecular Dynamic Simulation and Tensile Testing Experiment

In an attempt to address the issue of excessive bonding force between the mandrel and shell, we have devised an innovative solution by introducing a DLC (Diamond-Like Carbon) isolation film. Hence, it is imperative to validate our concept by conducting simulation calculations to assess the interface bonding force, as well as performing experimental tests to measure the interface bonding force. Molecular dynamics is employed to quantitatively assess the impact of Diamond-Like Carbon (DLC) on the interface energy.

### 3.1. Molecular Dynamic Simulation

#### 3.1.1. Calculation Method and Procedure

**Figure 6:**
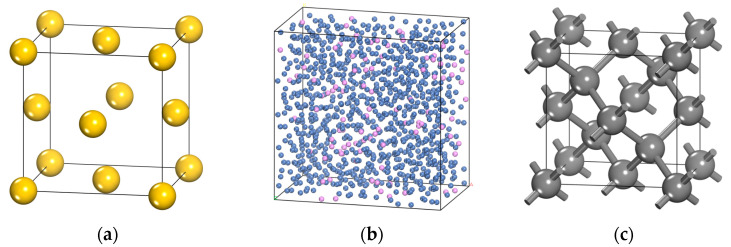
Au layer model (**a**), NiP layer model (**b**), C layer model (**c**).

The layers of Au, NiP, and C (where C represents DLC) are constructed in the following manner. The construction process for the Au layer model involves three steps: The construction of the Au cell is initiated. Based on the X-ray diffraction (XRD) findings reported by Dutta et al. [25], the gold (Au) cell space group is identified as FM-3M. The lattice parameters are determined to be a = b = c = 4.0783 Å, with the angles α = β = γ = 90°. The crystal model of Au is visually represented in Figure 6a. Then, the crystal face of the Au cell is cut. According to literature research, there are two representative crystal faces of Au: (100) and (111). Due to the crystal face of (111) having an angle of 120°, it presents challenges in matching the size and angle of the interface model. Therefore, this study opts to utilize the (100) crystal face for cutting. The Au (100) crystal surface is expanded to ensure that the dimensions of the Au layer obtained are greater than two times the cut-off radius of the van der Waals force used in the subsequent calculation (12.5 Å). Additionally, the thickness of the Au layer is required to be greater than one time the cut-off radius of the van der Waals force used in the subsequent calculation. The dimensions of the resulting Au layer model are 26 Å × 26 Å × 14 Å. The NiP is fabricated utilizing the Amorphous Cell (AC module) of the MS. The nickel-to-phosphorus (Ni/P) ratio has been established at a ratio of 9:1. The model is depicted in Figure 6b. To achieve a proper alignment with the Au layer, the NiP amorphous cell possesses identical length and width dimensions. However, the thickness of the NiP amorphous cell is slightly greater, measuring 16 Å.

The construction of the diamond layer model, similar to the Au layer model, is comprised of three distinct steps: The construction of the diamond cell is initiated. Based on the X-ray diffraction (XRD) experimental data presented by Straumanis et al. [26], the adamantine crystal cells exhibit a space group of FD-3M. The crystal structure parameters are as follows: the lattice parameters a, b, and c are equal to 3.556 Å, while the angles α, β, and γ are all 90°. The crystal model is visually depicted in Figure 6c. The crystal morphology of adamantine crystal cells was determined by utilizing the morphology module of MS software, employing the BFDH method [27,28,29]. Based on the calculation results, it has been determined that the morphology of diamond consists entirely of crystal faces belonging to the {111} crystal face group. This crystal face group corresponds to the hexagonal close-packed face of the crystal cell structure of adamantine. Specifically, there are eight equivalent crystal faces within this group: (1 1 1), (1 −1 1), (−1 1 1), (−1 −1 1), (1 −1 −1), (1 1 −1), (−1 −1 −1), and (−1 1 −1). It is worth noting that selecting any of these crystal faces for cutting will yield the same result. The (111) crystal plane has been chosen for cutting in this paper. The crystal surface is expanded to guarantee that the dimensions of the diamond layer obtained are greater than twice the cut-off radius of the van der Waals force used in the subsequent calculation (12.5 Å). Additionally, the thickness of the diamond layer is ensured to be greater than the cut-off radius of the van der Waals force used in the subsequent calculation. The dimensions of the final diamond layer model are 26 Å × 25 Å × 13 Å.

**Figure 7:**
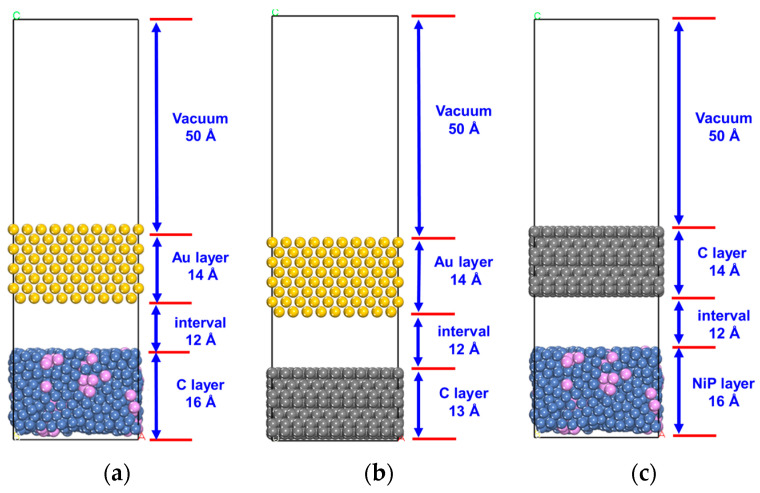
AU-NiP interface model (**a**), AU-C interface model (**b**), C-NiP interface model (**c**).

The resulting models are combined as follows: the Au and NiP layers are combined to form an AU-NiP interface model, as depicted in Figure 7a; the Au and diamond layer are combined to form an AU-C interface model, where C represents diamond, as shown in Figure 7b; and the NiP and diamond layer are combined to form a C-NiP interface model, as illustrated in Figure 7c.

#### 3.1.2. Calculation Parameter Setting

The configuration of energy calculation parameters is crucial for conducting adsorption calculations between interface models using the Forcite module. Among these parameters, the most significant ones pertain to energy calculation and encompass the configuration of the force field, van der Waals force, and electrostatic force. The parameters established in this paper are presented in Table 1.

Among the parameters mentioned earlier, the force field stands out as the most crucial. The acronym COMPASS stands for Condensed-phase Optimized Molecular Potentials for Atomistic Simulation Studies. The force fields in this series are derived using an ab initio algorithm fitting approach [30,31]. It has the advantages of high calculation precision and wide coverage. The COMPASS II force field combines quantum mechanical calculations (B3LYP/6-31G(d,p) accuracy level) to further improve the original COMPASS force field, which not only covers a wider range, but also has higher computational accuracy.

Molecular dynamics parameter setting is involved using the Forcite module of the MS software to perform a molecular dynamics simulation of the constructed interface model. The system is configured in the NVT ensemble, with the initial velocities following a Boltzmann random distribution. The simulated temperature is 298 K (25 °C), the thermostat is Andersen, the time step is 1 fs, the total simulation time is 500 ps, output a frame of the result configuration every 1 ps, for a total of 501 frames (including 1 frame of the initial configuration). The parameters governing the force field, including van der Waals force and electrostatic force, utilized in molecular dynamics simulations are established based on the corresponding parameters employed in energy calculations, as discussed earlier.

#### 3.1.3. System Balance Judgment

**Figure 8:**
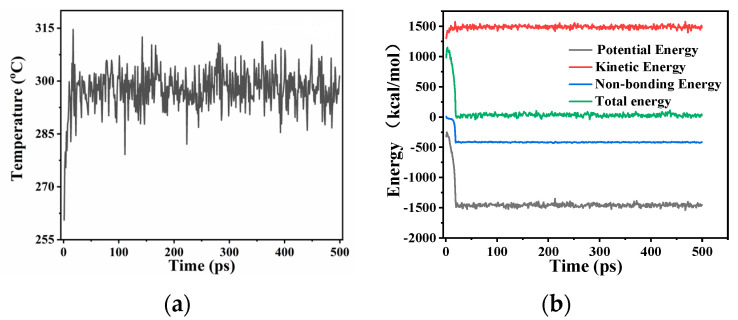
The change of temperature (**a**) and system energy over simulation (**b**).

The analysis of the results from molecular dynamics simulation should be conducted once the system has achieved equilibrium. Failing to do so may lead to obtaining unreliable or incorrect data. To ensure the reliability of the result, it is necessary to assess the balance of the system prior to conducting the result analysis. Figure 8a displays the temperature variation, while Figure 8b illustrates the system energy evolution throughout the simulation time. These figures serve as examples, utilizing the Au-C interface model. After approximately 100 picoseconds (ps), the temperature, kinetic energy, non-bonding energy, potential energy, and total energy of the system exhibit fluctuations around the equilibrium value. This behavior signifies that the system has achieved equilibrium following the 100 ps timeframe.

#### 3.1.4. Interface Energy Analysis

Once the system has been balanced, the calculation of the interfacial energy (*E_int_*) between two layers of substances within a given trajectory becomes possible. The formula for determining *E_int_* is as follows. When the value of *E_int_* is less than zero, it signifies the presence of adsorption between the two layers. Furthermore, a more negative *E_int_* value corresponds to a higher degree of adsorption, indicating a stronger adsorption phenomenon.

(1) $$E_{int}=E_{Layer\;1+Layer\;2}-\left(E_{Layer\;1}-E_{Layer\;2}\right)$$

In formula (1), *E_Layer_*_1+*Layer*2_ represents the energy of the entire interface model; *E_Layer_*_1_ represents the energy of a single Au or NiP layer; *E_Layer_*_2_ represents the energy of a single diamond layer. Formula (1) exclusively computes the interface energy associated with a specific frame configuration. The outcome derived from a particular frame configuration merely represents a single data point and lacks statistical significance in the broader context. Hence, it is advisable to select a substantial number of samples for the purpose of statistical analysis. According to the central limit theorem of statistics, when the number of samples is ≥30, the mean value of these samples can better reflect the property of the population. In this paper, there are 200 frames in total after 200 ps of molecular dynamics. The calculation of interface energy is performed for every frame, followed by statistical analysis. The average value of *E_int_* is then computed to quantify the adsorption strength between two layers of substances.

#### 3.1.5. Software and Modules

The research presented in this paper was conducted utilizing the open-source software Materials Studio (MS) 2020. The system primarily utilizes four modules, namely, MS Visualizer, Amorphous Cell, Morphology, and Forcite. The functionalities of the modules utilized in this study are presented in Table 2.

### 3.2. Tensile Testing Experiment

**Figure 9:**
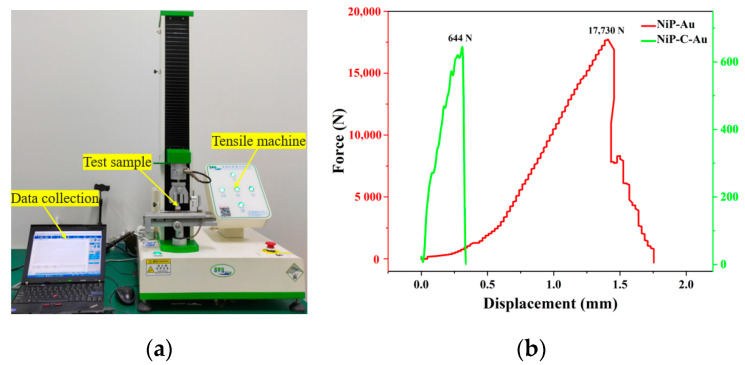
NiP-C-Au sample in tensile test (**a**), tensile test machine (**b**), Force–displacement curves of sample without DLC and with DLC.

The test sample consists of a substrate that has been chemically plated with NiP. Additionally, RF magnetron sputtering is used to deposit C and Au films onto the substrate. The thickness of the C film is approximately 20 nm, while the Au film has a thickness of approximately 150 nm. The power for Au deposition is 150 W, while the power for C deposition is 100 W. The target base distance is 200 mm. The film bonding force is tested by the adhesive peel method for flat specimens. The specimen size is φ20 mm cylindrical, and NiP-Au is tested by the tensile machine model CSS-44300 made by PT from Dong Guan city of China with the maximum force limit of 300 kN. The loading speed is 2 mm/min. Figure 9a illustrates the procedure for conducting a tensile force test on the sample. The test results indicate that the adhesion force between the Au layer and NiP without DLC is approximately 17,730 N, while the adhesion force between the Au layer and C is approximately 644 N. The force–displacement curves of the sample are depicted in Figure 9b.

### 3.3. Results and Discussions

The stability of the interface binding energy typically remains constant over time. Table 3 displays the computed average values of specific interface energies.

Based on the experimental findings, it can be observed that the fracture took place at the interface between the DLC and Au layers. Notably, the DLC film layer remained undamaged on the Nip substrates. The adhesion force between the Au layer and NiP was measured to be approximately 17,730 N, while the adhesion force between the Au layer and DLC was approximately 644 N. The simulation results exhibit a high level of agreement with the experimental results. The results presented above demonstrate a positive correlation between the interfacial energy value and the strength of interfacial adsorption, as well as the magnitude of the binding force. Simultaneously, the DLC release layer effectively safeguards the mandrel, enabling its reusability.

## 4. The Demolding System and Demolding Process

### 4.1. Demolding System

The mirrors are manufactured using the replication processing technique. During demolding, the mandrels and mirrors are separated, with the reflective surface located on the inner side of the mirrors. Hence, the detachment of the mirrors from the mandrels is an essential procedure. To address the limitations of existing methods, we have developed an automated demolding system. This system significantly enhances the consistency and controllability of the replication process, leading to reduced production cycles for focusing mirrors. Additionally, it improves replication efficiency, lowers labor costs, and offers flexible adjustments to meet the replication requirements of various sizes of focusing mirrors. The system exhibits high adaptability in accommodating different replication needs. The demolding device is used to remove the mirrors from the mandrels with no damage and to maintain the consistency of the mandrel with the mirrors’ shape as much as possible. The main operating principle of the demolding is that the thermal expansion coefficient of the aluminum mandrel is greater than the nickel mirrors. When the temperature of the mandrel is reduced, there is a contraction difference between the mirrors and the mandrel, resulting in the mirrors being separated from the mandrel. Once the mirror shell is released, it will be lifted by the demolding device immediately.

**Figure 10:**
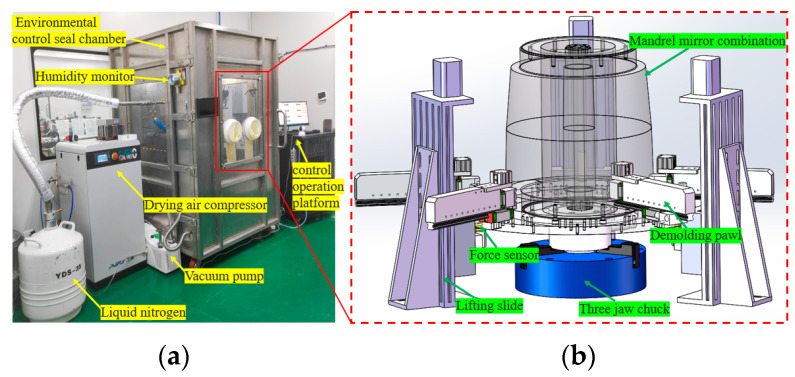
Overall layout of demolding system (**a**) and demolding device (**b**).

The demolding process necessitates the cooling of the mandrel and mirrors. The temperature required for cooling varies, depending on the size of the mandrel. In certain cases, the temperature may drop below freezing, causing water vapor in the air to condense on the mirror surfaces, resulting in frost formation. Therefore, it is essential to conduct the entire demolding process in an environment with minimal air humidity, ideally approaching zero. Therefore, the environmental control seal chamber is used to seal the entire demolding device, and the air compressor is used to continuously pour the dry air into the chamber to keep the positive pressure. The demolding system consists of several key components, including liquid nitrogen supply equipment, an environmental control seal chamber, a vacuum pump, a drying air compressor, a control operation platform, and a humidity monitor. These components are illustrated in Figure 10a. The demolding device consists of several key components, including the lifting slide, demolding pawl, force sensor, and three-jaw chuck. These components are illustrated in Figure 10b.

### 4.2. Demolding Process

To ensure effective monitoring and smooth control of the demolding process, three symmetrical high-precision mechanical sensors are strategically installed on the column of the vertical slide table. These sensors provide real-time feedback on the demolding force, allowing for timely adjustments to prevent excessive lifting force. By implementing this feedback mechanism, the stability of the demolding process is significantly enhanced.

According to the demolding principle, the demolding process is formulated as follows:

(a) Debug the demolding device and calibrate three mechanical sensors.
(b) Transfer the combination of the mandrel and mirror ready for release to the demolding device; adjust the size of the “step” in front of the demolding claw according to the thickness of different mirrors; then, move it to the lower end of the mirrors, while locking the demolding claw. Attach thermocouples to the upper and lower ends of the mandrel.
(c) Start the vacuum pump until the chamber humidity is lower than 5%, and continue to start the air compressor until the air humidity in the environmental control seal chamber drops to 1%; if the air source of the drying air compressor is not enough to make the internal air humidity lower, at this time, the way of passing high-purity nitrogen is further replaced until the air humidity is close to 0% (monitored by the humidity monitor).
(d) Open the self-pressurized liquid nitrogen tank and begin to continuously inject liquid nitrogen into the mandrel. Notice the gap between the edges of the mirror shell and the mandrel; stop liquid nitrogen as soon as the gap appears.
(e) At last, use the control operation platform to control the vertical sliding table to move upward slowly; always observe that the maximum value of the sensor indicator does not exceed 500 N. When the mirror shell is lifted higher than the mandrel, stop moving the vertical sliding table, remove the mirror shell, and the demolding is completed.

### 4.3. Demolding Experiment

**Figure 11:**
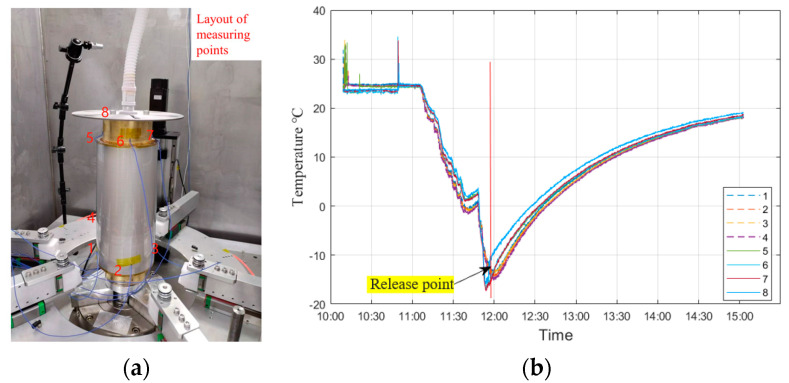
Temperature monitoring (**a**) and mirror shell release point temperature during demolding (**b**).

**Figure 12:**
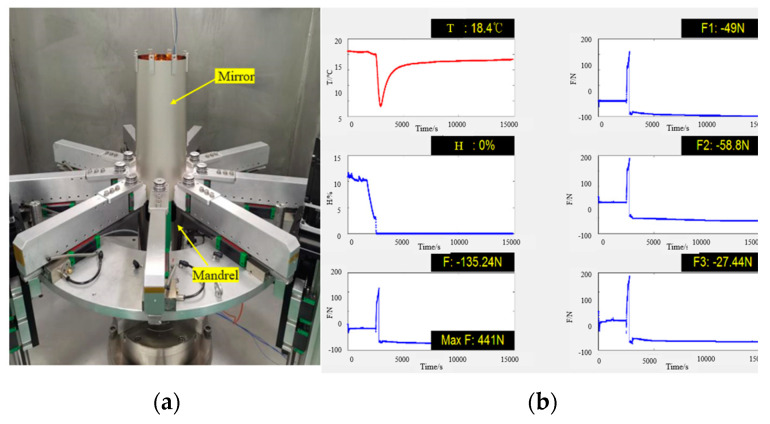
Lifting the mirror shell (**a**) and demolding force motoring (**b**).

The verification of the demolding process is conducted using experiment sample #32, which is produced through the electroforming replication process. Eight thermocouples were affixed onto #32. The temperature was subsequently lowered to approximately −16.2 °C, as depicted in Figure 11. At this temperature, the mirror shell successfully detached from the mandrel. The control operation platform is utilized to manipulate the vertical sliding table, thereby raising the mirror. During this process, the temperature within the cavity is recorded at 18.4 °C, while the humidity level remains at 0%. Additionally, the force sensor exhibits a maximum indicator reading of 441 N, as depicted in Figure 12.

### 4.4. Results and Discussions

**Figure 13:**
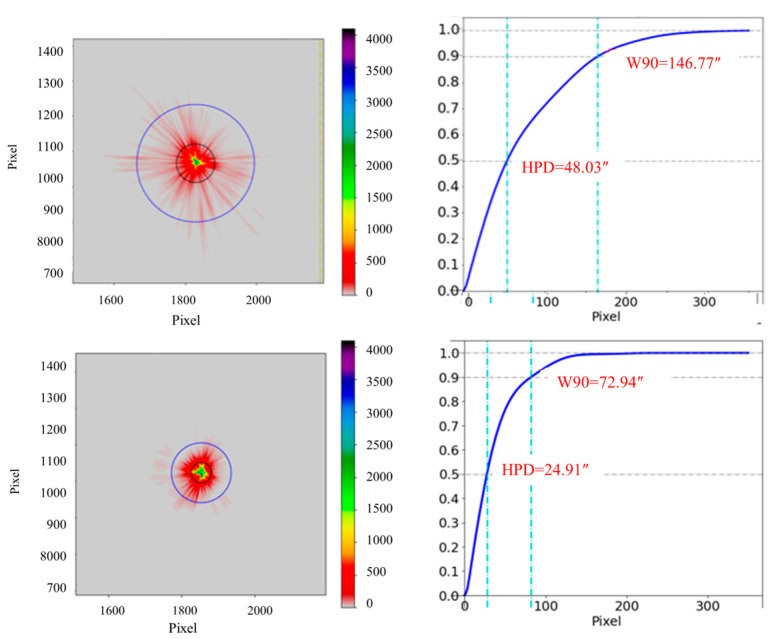
Focal spot and focal energy envelope function (EEF) of mirror 21 A without DLC release film (**top**) and 21 B with DLC release film (**bottom**).

The angular resolution is a crucial parameter for evaluating the performance of the focusing mirror. A smaller angular resolution indicates a higher level of mirror performance. In the case of X-ray focusing mirrors, the half-power diameter (HPD) is commonly employed to assess the angular resolution. Additionally, the defocus spot image of the mirrors and the appearance of the spot provide insights into the accuracy and roundness of the mirror’s surface. Figure 13 displays the focus spot and the function of focus energy enveloping (EEF).

The High-Performance Diameter (HPD) of the mirrors with Diamond-Like Carbon (DLC) release film applied on the mandrel surface is 24.9 inches, whereas the HPD of mirrors without DLC release film is 48.0 inches. This improvement is attributed to the effectiveness of the DLC release film in reducing the bonding between the mirrors and the mandrel. Consequently, the deformation of the mirrors during the release process is minimized, leading to an enhanced angular resolution.

**Figure 14:**
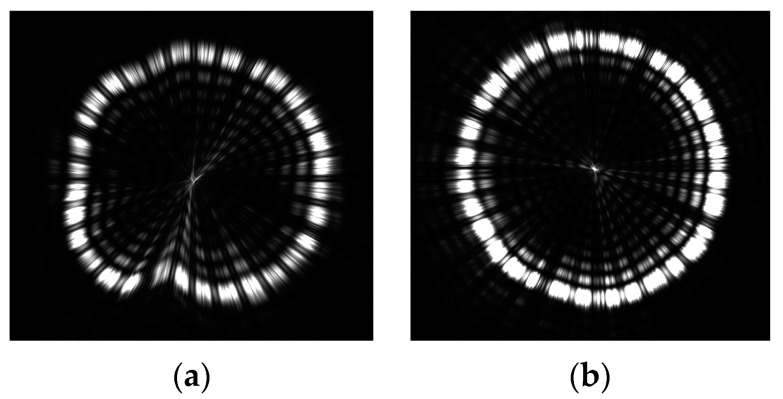
Defocusing spot test results of mirror without DLC film (**a**) and with DLC film (**b**).

Figure 14 illustrates the defocus spot test of the two mirrors. The roundness of mirrors with a DLC release film applied to the surface is superior to that of mirrors without DLC release. This is attributed to the reduced deformation experienced during the fabrication process. Therefore, the application of DLC release film has the potential to enhance the roundness of mirrors.

## 5. Conclusions

X-ray focusing mirrors are highly sought after in the domains of pulsar navigation, divine light engineering, space-based early warning, and advanced light sources within the space X-ray observatory system. The present research examines the fabrication process of X-ray focusing mirrors, specifically focusing on the introduction of a DLC release film to facilitate the release of the mirror shell from the mandrel. Additionally, a demolding system is developed to further enhance the performance of the X-ray focusing mirrors. Based on the available data and analysis, the following conclusions have been drawn:

1. We explored the optimized process of nickel electroforming replication, and the HPD of the mirror is reduced from 48″ to 25″, which is better than the production target.
2. Calculations and experiments based on molecular dynamics demonstrate that the interfacial energy value correlates to the interfacial adsorption strength and binding force.
3. The DLC release layer reduces the adhesion force of the film, facilitating the release of mirror shells and decreasing the danger of mirror distortion. The DLC release layer can also protect the mandrel, so that it can be reused.
4. To increase demolding efficiency and guarantee mirror quality, force-feedback automatic demolding equipment and a demolding process have been created.
5. The fabrication process of the X-ray focusing mirrors will provide key technical support for China’s space X-ray detection and contribute to X-ray astronomy.

## 6. Patents

Automatic reproduction device for large-size thin-wall X-ray focusing mirrors [P]. Heilongjiang Province: CN 113832505 B, 2022-04-08.

Automatic reproduction method of large-size thin-wall X-ray focusing mirrors [P]. Heilongjiang Province: CN 113913877 B, 2022-08-09.

## Figures and Tables

**Table 1:** Calculating parameter and Set result.

Calculating Parameter	Set Result
Force field	COMPASS II
Charge calculation method	Force field assigned
Van der Waals force calculation method	Atom based
Van der Waals force cutoff radius (A)	12.5
Electrostatic force calculation method	Ewald
Electrostatic force calculation accuracy (kcal/mol)	1 × 10^−3^

**Table 2:** The functions of the modules in this study.

Module	Function
MS Visualizer	Molecular, cell, interface model construction; model visualization and output
Amorphous Cell	The construction of the Au NiP and C cells
Morphology	The crystal morphology of the cell was calculated, and the representative crystal face was determined
Forcite	Molecular dynamics calculation and result analysis and output

**Table 3:** The calculated average values of specific interface energies.

Interface Model	*E_int_* (kcal/mol)
Au-NiP	−2843 ± 8
C-NiP	−595 ± 2
Au-C	−398 ± 2

## Data Availability

Not applicable.
